# Intranasal Immunization with Influenza VLPs Incorporating Membrane-Anchored Flagellin Induces Strong Heterosubtypic Protection

**DOI:** 10.1371/journal.pone.0013972

**Published:** 2010-11-29

**Authors:** Bao-Zhong Wang, Rui Xu, Fu-Shi Quan, Sang-Moo Kang, Li Wang, Richard W. Compans

**Affiliations:** Department of Microbiology and Immunology and Emory Vaccine Center, Emory University School of Medicine, Atlanta, Georgia, United States of America; Erasmus Medical Center, Netherlands

## Abstract

We demonstrated previously that the incorporation of a membrane-anchored form of flagellin into influenza virus-like particles (VLPs) improved the immunogenicity of VLPs significantly, inducing partially protective heterosubtypic immunity by intramuscular immunization. Because the efficacy of mucosal vaccination is highly dependent on an adjuvant, and is particularly effective for preventing mucosal infections such as influenza, we determined whether the membrane-anchored flagellin is an efficient adjuvant for VLP vaccines by a mucosal immunization route. We compared the adjuvant effect of membrane-anchored and soluble flagellins for immunization with influenza A/PR8 (H1N1) VLPs by the intranasal route in a mouse model. The results demonstrate that membrane-anchored flagellin is an effective adjuvant for intranasal (IN) immunization, inducing enhanced systemic and mucosal antibody responses. High cellular responses were also observed as shown by cytokine production in splenocyte cultures when stimulated with viral antigens. All mice immunized with flagellin-containing VLPs survived challenge with a high lethal dose of homologous virus as well as a high dose heterosubtypic virus challenge (40 LD_50_ of A/Philippines/82, H3N2). In contrast, no protection was observed with a standard HA/M1 VLP group upon heterosubtypic challenge. Soluble flagellin exhibited a moderate adjuvant effect when co-administered with VLPs by the mucosal route, as indicated by enhanced systemic and mucosal responses and partial heterosubtypic protection. The membrane-anchored form of flagellin incorporated together with antigen into influenza VLPs is effective as an adjuvant by the mucosal route and unlike standard VLPs, immunization with such chimeric VLPs elicits protective immunity to challenge with a distantly related influenza A virus.

## Introduction

Although most infectious pathogens enter through mucosal surfaces [1] traditional immunization strategies, including the parenteral route, do not induce effective mucosal responses [2], [3]. IN immunization has been shown to be effective for protection against infectious respiratory diseases such as influenza [4], [5]. Although there are attractive advantages of mucosal immunization over traditional injection routes, few of the current vaccines that are approved for human use are administered mucosally [6]. Often the effectiveness of mucosal immunization depends on co-administration of appropriate adjuvants that can initiate and support the transition from innate to adaptive immunity [7]. Mucosal adjuvants are required not only to boost mucosal and systemic immunity, but also to prevent the induction of mucosally induced tolerance [6]. Enterotoxins, including cholera toxin (CT) and heat-labile toxin (LT), have been very effective mucosal adjuvants experimentally, but their toxicity limits their use in humans [8]. Finding alternative mucosal adjuvants is therefore of high priority for the development of mucosal vaccines. The use of particulate antigens and adjuvants has been evaluated by several groups and found to be advantageous for mucosal immunization [9], [10]. Such particles (e.g., microparticles, virosomes, and virus-like particles [VLPs]) have comparable dimensions to pathogens that the immune system evolved to combat, and therefore are naturally targeted for uptake by antigen-presenting cells (APCs) to facilitate the induction of potent immune responses [11].

Influenza viruses are able to evade the host immune system since they continuously undergo antigenic evolution through the process of drift and shift [12]. Furthermore, poultry and migratory birds are reservoirs for new emerging influenza viruses which may cause pandemics in humans [13]. Although vaccination is the most effective approach to prevent influenza [14], [15], current influenza vaccines are highly strain-specific. Protection offered by the current inactivated influenza vaccines is mainly based on the induction of neutralizing antibodies against the surface protein hemagglutinin (HA). Novel influenza vaccines that induce a greater breadth of immunity may overcome limitations in vaccine efficacy in combating the antigenic variability of influenza A viruses [5].

Flagellin is the primary protein component of the highly complex flagellar structures that extend from the outer membranes of Gram-negative organisms. Flagellin has been shown to be recognized by TLR5, a member of the Toll-like receptor (TLR) families on mammalian cell surfaces [16]. Acting as the natural agonist of TLR5, flagellin is a strong inducer of innate immune effectors such as cytokines and nitric oxide [17], [18] and is a potent and effective adjuvant [19], [20]. Because mucosal immunization offers many attractive features compared with other routes in prevention of mucosal infection, and influenza VLPs are a potent new generation of vaccines, we determined whether mucosal immunization with influenza VLPs containing membrane-bound flagellin induces enhanced immune responses, including mucosal and systemic responses with broad reactivity.

## Results

### IN immunization with flagellin-containing influenza VLPs induces strong mucosal responses

**Figure 1:**
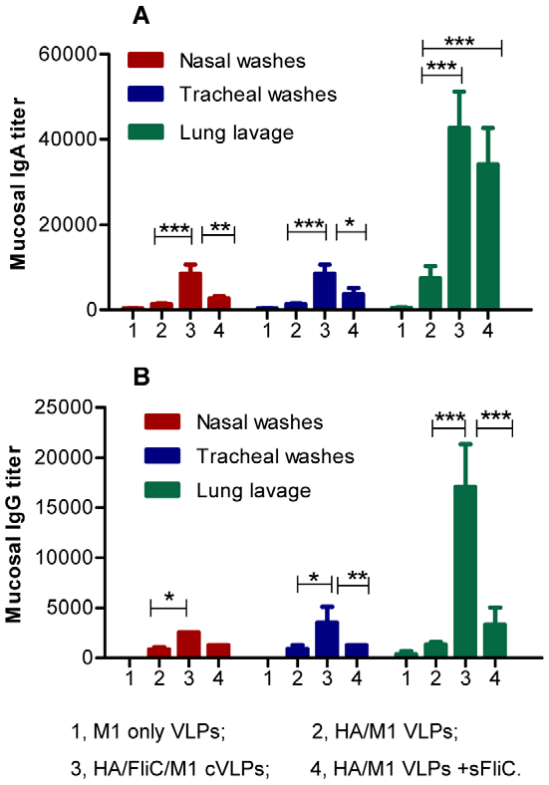
Mucosal IgA and IgG titers against PR8 virus. Samples were collected as described in Materials and Methods. Collected mucosal samples were cleared by a brief centrifugation (8000 rpm for 10 min), and assessed by ELISA. Microtiter plates were coated with 100 µl/well of inactivated PR8 virus (5 µg/ml) and HRP-conjugated goat anti-mouse IgA or IgG antibodies were used for detection. Antibody endpoint titers were defined as the highest serum dilution (fold) which gave an OD450 value 2-fold higher than naïve mice at the lowest dilution (200×). Representative data are the geometric mean ± standard deviation (SD) of six mice per group. Comparisons were performed using the non-matching two-way ANOVA followed by Bonferroni's post test. P<0.05(*), P<0.01(**), P<0.001(***), P>0.05(n.s.). **A**, Mucosal IgA titers; **B**, Mucosal IgG titers. Bars 1, 2, 3 and 4 presented groups of M1 only VLPs, HA/M1VLPs, HA/FliC/M1 cVLPs, and HA/M1VLPs + sFliC, respectively. FliC, flagellin; sFliC, soluble flagellin.

It is well recognized that mucosal immune responses are effective for protection against diseases initiated by mucosal surface infection [21]. These immune responses are most efficiently induced by the direct application of vaccines onto mucosal surfaces, and are enhanced by co-administered adjuvants [6], [22]. To determine whether membrane-anchored flagellin functions as a mucosal adjuvant and induces enhanced mucosal antigen-specific responses when incorporated into influenza VLPs, mice were immunized IN with defined doses of standard influenza VLPs (HA/M1 VLPs), chimeric VLPs (HA/FliC/M1 cVLPs), a mixture of standard VLPs with recombinant soluble flagellin (HA/M1 VLPs + sFliC), or M1 VLPs (Table 1), as described in Materials and Methods. The flagellin-containing VLP-immunized mice showed IgA (Fig. 1A) and IgG (Fig. 1B) titers of 4.3×10^4^ and 1.6×10^4^ in the lung lavage, which were 5.7- and 13-fold higher than those of the standard VLP group (7.5×10^3^ and 1.2×10^3^ respectively). Although the IgA and IgG levels in nasal and tracheal washes were lower compared to those in mouse lung lavage, the membrane-anchored flagellin-containing VLP-immunized group showed about 6.4-fold higher IgA levels in both nasal and tracheal washes (8.3×10^3^), and 2.8- and 4-fold higher IgG levels in nasal (2.6×10^3^) and tracheal (3.6×10^3^) washes compared to those of mice immunized by standard influenza VLPs. Recombinant soluble flagellin also showed a mucosal adjuvant effect when co-administered with influenza VLPs but its activity was lower than that of the membrane-anchored form. As shown in Fig. 1A, the IgA levels in the lung lavage, nasal and tracheal washes of mice immunized with the mixture of recombinant flagellin and standard VLPs were lower than those observed with flagellin-containing VLPs but were higher than those of standard VLP-immunized mice. The IgG titer in the lung lavage of mice immunized by the mixture of soluble flagellin and standard VLPs was 3.3×10^3^, 2.5-fold higher than the standard VLP group, but the IgG titers in nasal and tracheal washes of these mice did not show a significant difference from that of the standard VLP group (Fig. 1B).

**Table 1:** Mouse groups for IN immunization.

Groups*	Dose**
VLPs	10 µg VLPs
cVLPs***	10 µg VLPs
VLPs + sFliC****	10 µg VLPs+0.5 µg sFliC
M1 only VLPs	10 µg VLPs

*Naïve mice and a PBS immunized group did not show antigen specific responses and thus are not presented here.

**Doses were given in a total of 25 µl split between two nostrils.

***The dose of cVLPs was normalized to contain an HA content equal to 10 µg of standard VLPs by Western Blot. The content of the flagellin protein was 0.5 µg in each dose of cVLPs calculated by using purified recombinant flagellin as a standard.

****Purified recombinant soluble flagellin (sFliC) was mixed with standard VLPs before administration.

To determine whether the mucosal secretions have neutralization activity, the ability of these mucosal samples to neutralize live PR8 virus was evaluated using a MDCK cell-based plaque assay. As shown in Table 2, the neutralization titers of mice immunized with VLPs containing membrane-anchored flagellin were 80, 20 and 20 in lung lavage, nasal and tracheal washes, respectively. In contrast, mice immunized with standard VLPs, or a mixture of soluble flagellin and standard VLPs, showed a neutralization activity of 40 in lung lavage, and no activity in nasal or tracheal washes. We further evaluated whether the mucosal samples neutralize a heterosubtypic virus, A/Philippines/82 (H3N2), and found that lung lavage from the cVLP group showed the highest functional neutralization titer of 20. All other mucosal samples showed lower or undetectable neutralization titers to the Philippines virus. The results demonstrate that membrane-anchored flagellin enhances the immunogenicity of VLPs when immunized mucosally, inducing high levels of mucosal immunity exhibiting broadened reactivity.

**Table 2:** Neutralization activity of mucosal samples using A/PR8 virus.

Groups	Lung lavage	Nasal washes	Tracheal washes
VLPs	40*	<10	<10
cVLPs	80	20	20
VLPs + sFliC	40	<10	<10
M1 VLPs	undetectable	undetectable	undetectable

*Number represents fold dilution of mucosal samples which induced 50% plaque reduction. Six mice were included in each group. Data represents means of six mice.

### Chimeric VLPs containing membrane-anchored flagellin elicit high levels of systemic antibody responses by the IN route

**Figure 2:**
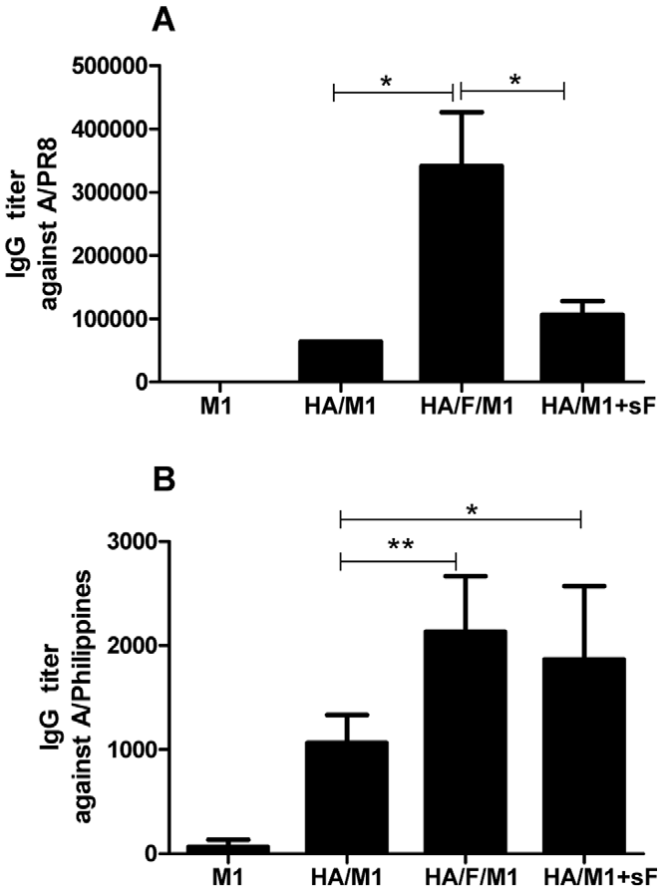
Serum IgG endpoint titers. Endpoint titers of immune serum IgG specific to A/PR8 (**A**) or cross-reactive binding to A/Philippines (**B**) were determined using ELISA as described in Materials and Methods. HRP-conjugated goat anti-mouse IgG antibody was used for detection. Endpoint titers were defined as in Fig 1. M1, M1 only VLPs; HA/M1, HA/M1 VLPs; HA/F/M1, HA/flagellin (FliC, F)/M1 cVLPs; HA/M1+sF, mixture of HA/M1 VLPs plus soluble flagellin (sFliC, sF).

An advantage of mucosal over parenteral immunization is the ability to induce both systemic and mucosal antigen-specific responses. To determine if IN immunization with flagellin-containing influenza cVLPs induced enhanced humoral responses, the influenza-specific IgG endpoint titers of immune sera were evaluated. As shown in Fig. 2A, more than 5-fold higher virus-specific serum IgG levels were observed in mice immunized with membrane-anchored flagellin-containing cVLPs (3.4×10^5^) compared with the standard influenza VLP group (6.4×10^4^). When recombinant flagellin was mixed and co-administered with standard VLPs, systemic responses were enhanced but to a lesser extent than observed with cVLPs. These results indicate that the membrane-anchored flagellin shows a stronger adjuvant effect than the soluble form in induction of serum IgG responses.

We also evaluated whether serum antibodies induced by cVLPs exhibit higher heterosubtypic reactivity. As shown in Fig. 2B, the serum IgG endpoint titer of the cVLP (HA/FliC/M1) group specific to Philippines virus (H3N2) was 2-fold higher than that of the standard VLP group (HA/M1 VLPs). Similar to the membrane-anchored form, soluble recombinant flagellin also showed significant enhancement of humoral heterosubtypic reactivity when co-administered with VLPs (group HA/M1 VLPs+sFliC), with 1.8-fold higher antibody binding to Philippines than the standard VLPs. These results demonstrate that both the membrane-anchored flagellin and the soluble form are effective in inducing enhanced systemic antibody responses exhibiting heterosubtypic binding activity.

**Figure 3:**
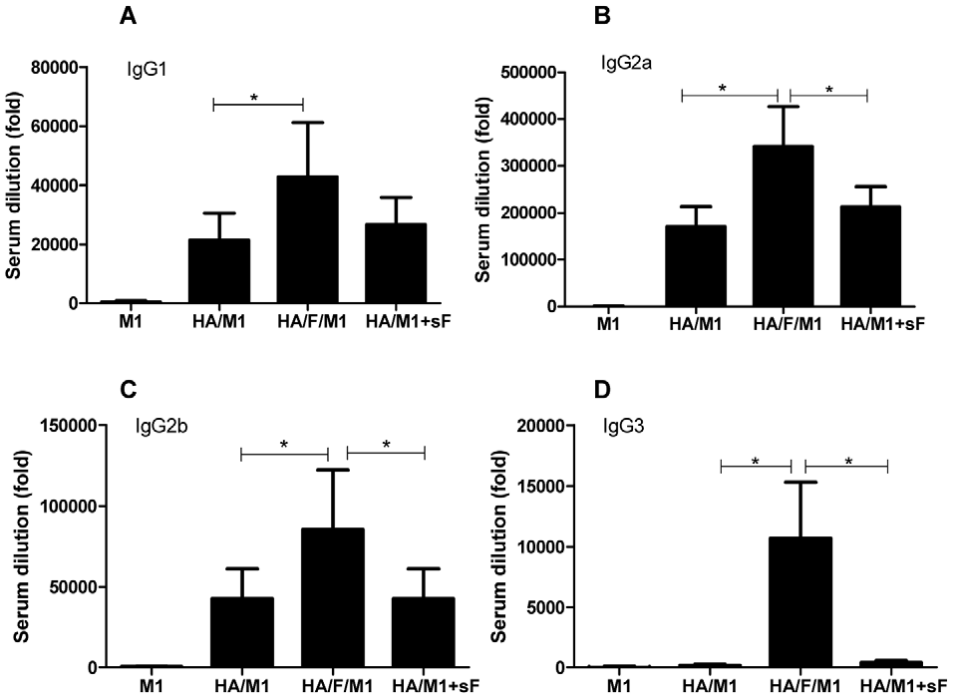
Endpoint titers of PR8-specific IgG subclasses. IgG subclass endpoint titers were determined as for serum IgG endpoint titer but using HRP-conjugated goat anti-mouse IgG1(A), IgG2a (B), IgG2b (C) or IgG3 (D) for detection.

B lymphocytes can change the isotype and subclass of the antibody they express by immunoglobulin (Ig) isotype switch recombination. The Ig isotype profile reflects different mechanisms of antigen presentation and cognate interaction with helper T cells. To understand how IgG subclasses are affected by the presence of the membrane-anchored flagellin in VLPs, the serum PR8-specific IgG subclass endpoint titers of immunized mice were assessed and compared to those of mice immunized with standard VLPs, or mixture of standard VLPs and recombinant flagellin. As shown in Fig. 3, the membrane-anchored flagellin boosted the production of all subclasses compared to the standard VLP group with/without soluble recombinant flagellin. In particular, cVLPs induced high levels of the IgG3 isotype while the standard VLPs or the mixture of the standard VLPs and recombinant flagellin showed IgG3 at background levels as shown in Fig. 3D. It is known that IgG1 and IgG3 subclasses can directly recruit phagocytic cells to ingest antigen-antibody complexes, as well as activating the complement system [23]. These results demonstrate that the membrane-anchored flagellin in cVLPs not only improves the antibody production quantitatively but also qualitatively changes the subclasses of antibodies induced, indicating different activation mechanisms of B cells.

### Membrane-anchored flagellin induces serum antibody responses conferring enhanced hemagglutination inhibition (HI) and virus neutralization

**Figure 4:**
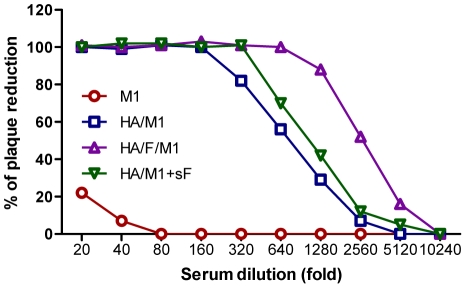
Neutralization activity against PR8. Immune sera were diluted 2-fold stepwise and incubated with virus (100 PFU) at 37°C for 1 h. A standard plaque reduction assay was performed using MDCK cell cultures.

As described above, cVLPs containing the membrane-anchored flagellin enhanced both mucosal and systemic antibody responses. We further determined whether cVLPs induce serum antibodies with higher neutralizing titer (NT) in a MDCK-based neutralization assay. As showed in Fig. 4, the cVLP group showed a NT of 2560, which was 4-fold higher than that of the standard VLP group (640), demonstrating the adjuvant effect of the membrane-anchored flagellin for inducing antibodies which are functional in blocking infection by live virus. In contrast to cVLPs, the mixture of soluble flagellin and standard VLPs induced a moderately enhanced NT (1280). However, the above groups did not show detectable NT to the A/Philippines virus (data not shown).

**Figure 5:**
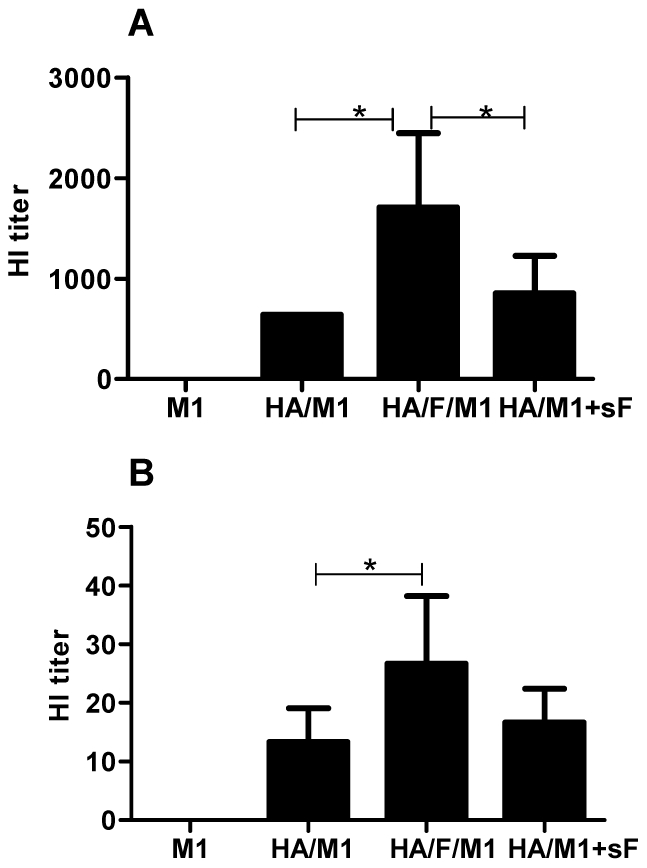
Hemagglutination inhibition (HI) titers against H1N1 PR8 or H3N2 Philippines virus. **A**, HI titers to PR8 virus; **B**, HI titers to Philippines virus. HI titers of immune sera were determined as the capacity of sera to inhibit virus hemagglutination of chicken red blood cells. Representative data are the geometric mean ± S.D.

For influenza vaccines, the induction of antibodies with hemagglutination-inhibition (HI) capacity correlates with their protective efficacy. To further compare the systemic responses induced, we determined the HI activity of immunized mouse sera against both A/PR8 and A/Philippines viruses. As shown in Fig. 5A, the cVLPs group induced HI responses to A/PR8 virus at a mean titer of 1700, while the responses to the standard VLP group were 640. The mixture of soluble flagellin with standard VLPs induced HI titers with a mean value of 850. As shown in Fig. 5B, cVLPs containing the membrane-anchored flagellin also induced antibodies showing HI activity to A/Philippines virus (a mean value of 28). In contrast, both standard VLPs and the mixture of soluble flagellin with standard VLPs showed HI titers less than 20. These results further demonstrate that the membrane-anchored flagellin incorporated together with antigen in the same VLPs is effective as a mucosal adjuvant.

### Membrane-anchored flagellin promotes antigen-specific T cell responses

**Figure 6:**
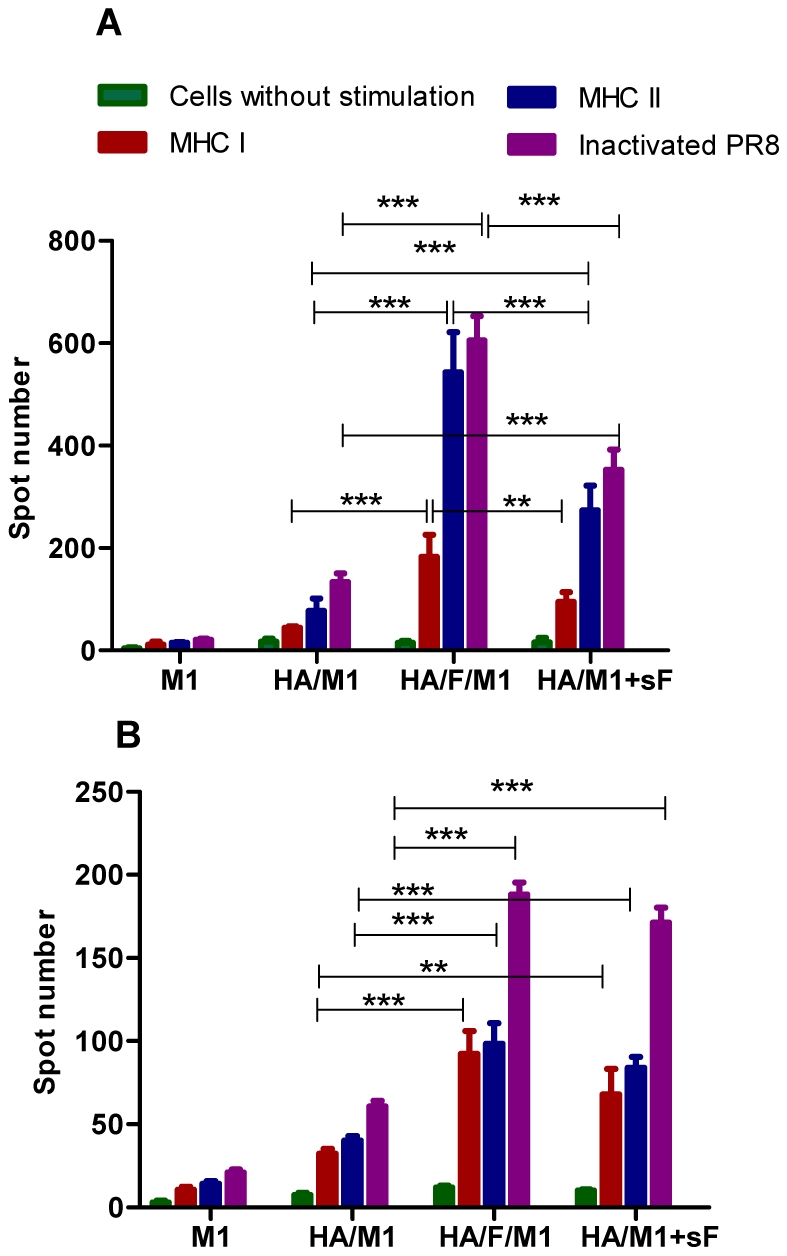
Cytokine secretion from immunized mouse splenocytes. ELIspot assays were used to detect the proliferation of antigen-specific CD4+ secreting INF-γ (**A**) or IL-4 (**B**) cells as described in “Materials and Methods”. Splenocytes were isolated from immunized mice 4 weeks after the boosting immunization and seeded into pre-coated Multiscreen plates (Millipore). Defined peptides (MHC I or MHC II) or inactivated PR8 viruses were added to stimulate antigen-specific T cell proliferation. Cytokine secreting clones were visualized by spot staining following the manufacturer's instruction. Representative data were mean ± S.D. of spot number in 1×10^6^ splenocytes of six mice.

Antigen-specific T lymphocytes are important for the generation and regulation of an effective immune response. Cytokines produced by T cells upon antigen stimulation have a central role in these processes. To determine whether membrane-anchored flagellin-containing VLPs induce different cytokine patterns, which may up-regulate antigen-specific immune responses, INF-γ or IL-4-producing T lymphocyte splenocyte populations were evaluated using ELISpot assays. As shown in Fig. 6, cVLP-immunized mice had the highest INF-γ and IL-4 producing populations compared to other groups when stimulated with MHC I or II-restricted PR8 HA peptide pools, revealing both high antigen-specific Th1 and Th2 subpopulations of helper T cells (CD4+). Soluble flagellin, used in the same amount, induced a weak enhancement of the INF-γ producing population as shown in Fig.6A and a moderate enhancement of the IL-4 producing population (Fig. 6B). These results demonstrate that membrane-anchored flagellin enhances the magnitude of cellular responses to influenza VLPs.

### Membrane-anchored flagellin enhances heterosubtypic protection against lethal virus challenge

**Figure 7:**
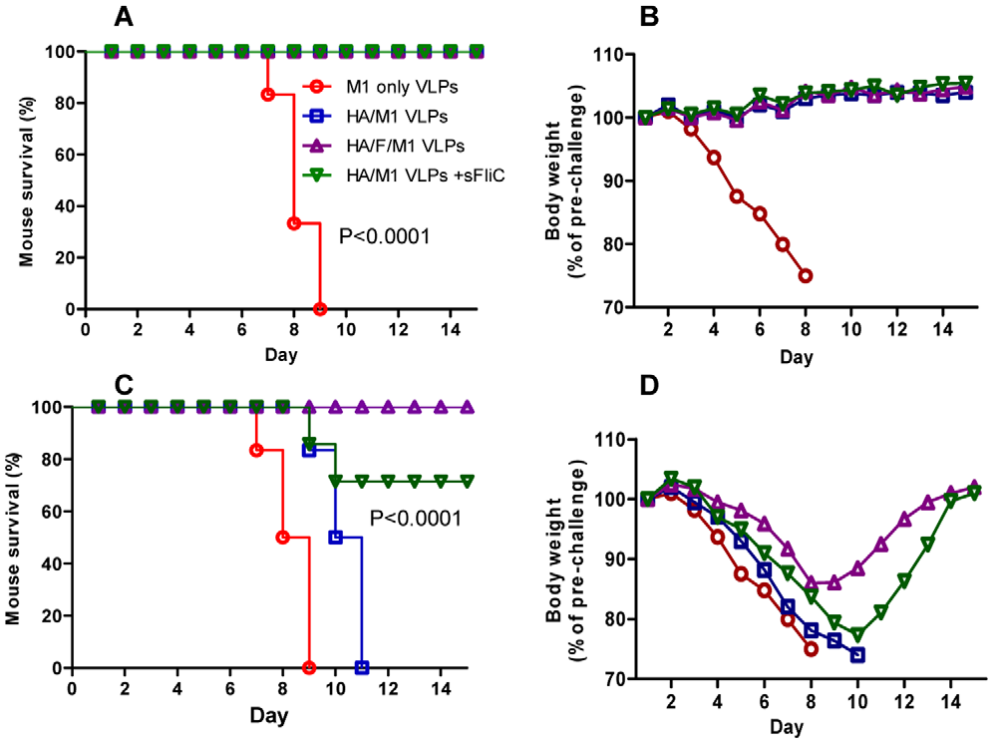
Live virus challenge using A/PR8 (H1N1) or A/Philippines (H3N2) viruses. Immunized mice were challenged with a lethal dose (40 LD_50_) of A/PR8 (**A** and **B**) or A/Philippines (**C** and **D**) in a volume of 25 µl by IN instillation. Mouse survival (**A**, **C**) and body weight changes (**B**, **D**) were monitored daily.

To determine whether the enhanced immune responses which we observed with cVLPs correlate with protective efficacy against virus infection, immunized mice were challenged with lethal doses of the homologous H1N1 PR8 virus (40 LD_50_) or the heterosubtypic H3N2 Philippines virus (40 LD_50_). For PR8 virus challenge, all mice were protected by VLP immunization except for the M1 VLP group (Fig. 7A) as expected. Also, no clinical sign of illness was observed as monitored by mouse body weight change (Fig.7B), demonstrating the efficacy of HA-containing VLPs in inducing protective immunity to the homologous virus. However, the standard influenza VLPs did not provide protection against the heterosubtypic virus. All mice infected with Philippines virus (40 LD_50_) reached their endpoint (25% of body-weight loss), as observed with the M1 only VLP group (Fig. 7C). The mixture of standard influenza VLPs with soluble flagellin provided partial protection, demonstrated by survival of 4 of 6 mice (67%) and 22% body-weight loss. In contrast, the membrane-anchored flagellin-containing VLPs provided 100% protection to the Philippines virus challenge (Fig. 7C), and only a moderate weight loss was observed (Fig. 7D). These results indicate that flagellin together with antigen in the same particulate structure functions better as a mucosal adjuvant as shown by the increased breadth of protective immunity.

**Figure 8:**
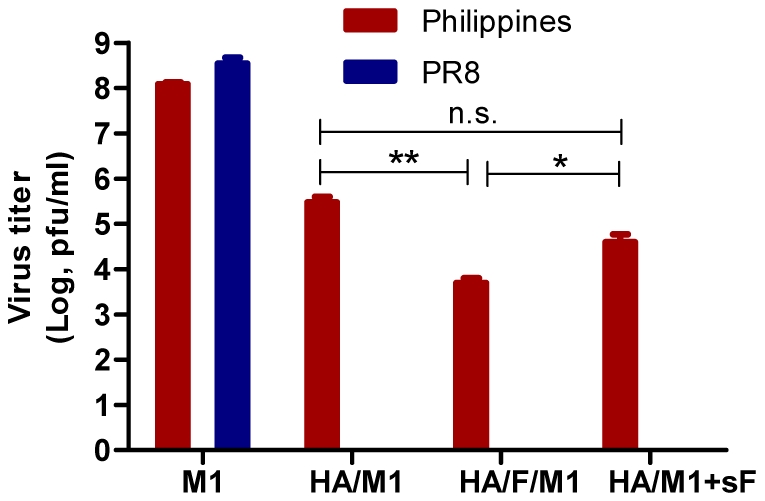
Lung viral loads on day 4 post-challenge infection with the homologous virus A/PR8 or the heterosubtypic virus A/Philippines. Mice immunized IN with designed doses of VLPs were challenged with a lethal dose (40 LD_50_) of A/PR8 or A/Philippines. Six mice were included in each viral challenge group. Four days after infection, mouse lungs were collected and extracted with medium RPMI 1640. The extract was adjusted to 1 ml per lung. Virus titers in lung extracts were titrated using MDCK-based plaque assay as described in Materials and Methods. The lung virus titer is presented as pfu per lung.

Lung viral loads are important measures of decreased morbidity and mortality after infection. Thus we determined the virus loads at day 4 in the lungs of immunized mice after challenge infection with PR8 or Philippines viruses. As shown in Fig. 8, all HA-containing VLPs were effective in clearance of PR8 virus, indicated by undetectable lung virus titers on day 4 in comparison to the high virus load of the control group (M1 only VLP group, 8.4×10^8^ PFU/lung). For A/Philippines virus-infected mice, the standard VLP group showed a virus titer of 3×10^5^ PFU/lung, whereas the cVLP group showed 60-fold lower titers of 5×10^3^. The mixture of standard VLP with soluble flagellin was 4×10^4^ PFU/lung, 8-fold lower than the standard VLP group. Thus, IN immunization with influenza cVLPs is more effective at inducing protective immunity against a heterosubtypic virus challenge.

## Discussion

VLPs have been tested as mucosal vaccines to induce protective immunity against mucosally transmitted infectious diseases [24], [25]. Because of the advantages of mucosal immunization and the enhancement of the mucosal immune responses by adjuvants [21], it was of high interest to determine whether a membrane-anchored TLR agonist can function as mucosal adjuvant when incorporated into VLPs. The current studies were therefore designed to determine the responses to chimeric flagellin-containing VLPs as influenza vaccines using an IN immunization route. An important characteristic of the mucosal adaptive immune responses is the local production and secretion of dimeric or multimeric IgA antibodies that are resistant to degradation in the protease-rich mucosal environment[26]. Our results show that influenza cVLPs incorporating flagellin induced high levels of IgA and IgG secretion and enhanced mucosal virus neutralization activity against homo- or heterosubtypic viruses compared with standard VLPs or a mixture of standard VLPs with soluble flagellin. The same amount of flagellin in soluble form showed a detectable but reduced adjuvant effect in boosting mucosal IgA secretion, suggesting that different mechanisms may be involved in sensing these two different forms. It is known that APCs can be activated by flagellin [19], and these cells consequentially control the direction of the induced immune responses. The upper respiratory tract (nasal-associated lymphoid tissue, NALT) contains an abundance of APCs, in particular mucosal DCs, expressing TLR molecules on their surfaces [2], [27]. Flagellin in cVLPs binds to TLR5 and thus not only can initiate innate signaling to secrete cytokines and upregulate other immune cells but also may facilitate DCs to ingest antigens and then present them to helper T cells [11], [28]. In contrast, co-administered soluble flagellin may target different DCs from those which encounter VLP antigens. The incorporation of membrane-anchored flagellin into the VLPs ensures that the antigen and adjuvant are delivered to the same cells, which may provide the basis for the much higher adjuvant effects of the flagellin incorporated in VLPs compared with the soluble form when used in the same amount.

An effective mucosal vaccine should induce both mucosal and systemic immune responses. Our results show that the membrane-anchored flagellin-containing VLPs induce enhanced antigen-specific systemic immunity. This enhancement was illustrated by higher levels of serum antibody titers with improved neutralization activity and HI titers. Increased systemic responses have been reported to be promoted by different forms of flagellin when mucosally administered, including polymeric, recombinant soluble, or fusion proteins and truncated flagellins [20], [29], [30]. A notable finding in our study was that the membrane-anchored flagellin co-incorporated with antigen into VLPs was more effective in inducing more broadly reactive antibody responses compared to soluble recombinant flagellin. Although soluble flagellin also induced enhanced antibody responses (HAI titers) to the homologous H1 virus when compared to standard VLPs, the enhancement of HAI titers to a heterologous H3 virus was not significant. These results suggest that membrane-anchored flagellin is more effective in boosting responses directed against cross-reactive determinants shared in HA molecules of distantly related heterosubtypic strains. Since the HAI titer is known to be correlated with protection in influenza vaccination, these results also support the conclusion that the enhanced antibody responses contribute to the observed cross-protection. We found previously that membrane-anchored flagellin enhanced immunity and conferred partial heterosubtypic cross-protection by intramuscular immunization [31]. The present data show that cVLPs containing flagellin are able to confer complete heterosubtypic protection by the IN route, indicating that the incorporation of flagellin into VLPs as mucosal adjuvant has great potential for developing broadly cross-reactive influenza vaccines.

A potentially new pandemic influenza virus, and particularly a highly pathogenic strain of a novel subtype, will likely allow little time for the development and production of a traditional strain-specific vaccine. Increased breadth of immunity with cross-protection against drifted and even shifted strains has long been pursued in the development of influenza vaccines because of the potential to prevent epidemics and possible pandemics [32], [33]. Although there is high genetic diversity of the HA peptides between the different subtypes, certain regions of the HA molecules of antigenically different subtypes show considerable sequence homologies which are attributed to the need for conservation of function and structural requirements, in particular in the HA2 portion and more deeply located regions of the HA1. An overall conservation of 33–36% of amino acid sequences between HAs has been reported [34]. PR8/34(H1N1, HA sequence access no. AA67033) and Philippines/2/82 (H3N2, AH sequence access no. ABQ58936.1) share 34% amino acids in their peptides when compared using MegAlign 7 (DNASTAR Inc., Madison, WI). Such similarity in the HA molecules could provide a basis for the observed cross-protection. Several strategies have been used to confer enhanced protection including the use of adjuvants [35], [36], [37]. With inactivated vaccines the most effective cross-protective immunity was achieved by IN delivery with adjuvants [5], [38], [39]. The results in the present study indicate that besides providing enhanced immunity against the homologous subtype, cVLPs containing the membrane-anchored flagellin induce protective immunity against heterosubtypic challenge when a H3N2 virus was used, whereas standard influenza VLPs did not confer any detectable heterosubtypic protection. Although a significant role of cellular immune responses in providing heterosubtypic protection has been reported with adjuvanted inactivated viruses or cold-adapted live virus vaccines [5], [37], [39], cell-mediated immunity may not be an important effector in heterosubtypic responses induced by our chimeric VLPs because few shared T cell epitopes are found in their HA and M1 protein components. However, significantly enhanced cellular immune responses against homologous antigens were induced by IN delivery of flagellin-containing VLPs as demonstrated by the recall of antigen-specific cytokine release, in particular INF-γ production, by splenocytes from immunized mice. INF-γ stimulates the expression of both MHC I and II molecules and co-stimulatory molecules on APCs, and promotes the differentiation of naïve helper T cells into Th1-biasing immunity CTL responses [40]. Although nonspecific cells including NK cells and nonlymphoid cell types such as macrophages in raw splenocytes are potential sources of cytokines, these cell types may not contribute to antigen-specific cytokine production. These observations demonstrate that mucosal delivery of cVLPs containing flagellin enhances both humoral and cellular immune responses and thus may therefore present a valuable approach in controlling new influenza epidemics and pandemics.

A broadly cross-protective influenza vaccine would have at least two potential advantages. One of these is to increase the breadth of immunity against new seasonal variants (drift strains), thus reducing the need for annual revaccination. Because of recent concerns about vaccine adjuvants, and the variable impact of such seasonal variants, the flagellin-containing VLPs may not be accepted for such use at least in some countries, although we did not observed any symptoms of illness when a dose 0.5 µg flagellin per mouse was used by IN immunization. A second major advantage of a broadly cross-protective influenza vaccine is to control a newly emerging pandemic strain, against which the population has no immunity. Such a vaccine could be produced in advance, and be rapidly deployed to limit the spread and the disease severity resulting from a new pandemic virus. In the event that such a pandemic virus strain causes a highly lethal human infection, such as those caused by avian H5N1 viruses, the rapid deployment of an adjuvant-containing VLP vaccine could have a major impact on reducing morbidity and mortality.

## Materials and Methods

### Ethics Statement

Mice were sterile housed and treated according to Emory University (Atlanta, GA) guidelines and all animal studies were approved by the Emory University Institutional Animal Care and Use Committee. Emory University's Animal Welfare Assurance number is A3180-01.

### Cell lines and viruses

*Spodoptera frugiperda* Sf9 cells (Sf9, ATCC, CRL-1711) and Madin-Darby canine kidney (MDCK, ATCC, CCL-34) cells were cultured as described previously [31]. Influenza viruses A/PR8/34 (H1N1) and A/Philippines/2/82 (H3N2) were grown in 10-day-old embryonated hen's eggs and purified from allantoic fluid by using a discontinuous sucrose gradient (15%, 35%, and 60%) [39]. Inactivation of the purified viruses was performed by adding formalin to a final concentration of 1∶4000 (v/v) as described [41], [42]. Mouse-adapted PR8 and Philippines viruses were prepared as lung homogenates from intranasally infected mice as described [39]. The mouse-adapted viruses were titrated by infection of mice with various dilution, and the LD_50_ was calculated by the method of Reed and Muench [43].

### VLP production and characterization

Recombinant baculoviruses (rBVs) and VLPs were produced as described [31]. In brief, rBVs expressing PR8 HA, flagellin (FliC), or M1 were propagated in Sf9 cell culture, and their titers were determined by plaque assay. Standard influenza VLPs (HA/M1 VLPs) and flagellin-containing influenza chimeric VLPs (HA/FliC/M1 cVLPs), as well as VLPs containing M1 only, were produced by co-infection of Sf9 cells with recombinant baculoviruses expressing HA and M1, FliC, HA and M1, or M1 only, respectively. For cVLPs, the MOIs of rBV expressing HA, M1 and FliC were adjusted to 2∶6∶3 to produce cVLPs containing 5% flagellin. The quality of purified VLPs was determined by Western blotting analysis, hemagglutination activity analysis, and electron microscopic observation [31]. The sterility was determined by inoculation into LB and culturing at 37°C for 48 h. The flagellin content in cVLPs was evaluated using ELISA as described [31].

### Mouse IN immunization

Inbred female BALB/c mice were obtained from Charles River Laboratory. Six-mouse groups were IN immunized twice at 4-week intervals (weeks 1 and 5). To perform IN immunization, mice were lightly anesthetized by inhalation of isofluorane and a total of 25 µl containing defined doses of VLPs and flagellin (Table 1) was instilled into the nostrils. VLP concentrations of 10 µg were used because previous studies have shown that this dose can induce protective immunity [31], [44]. We found that mice were more sensitive to flagellin by the IN immunization, therefore 0.5 µg flagellin was used in the IN immunization compared to 0.8 µg flagellin used in previous experiments for intramuscular immunization [31].

### Sample collection

Blood samples were collected by retro-orbital plexus puncture. Sera were collected by a brief spin (5000 rpm for 5 min) after clotting (about 1 hour at room temperature) one week before immunization (pre-immune sera, week 0) and 4 weeks after the boosting immunization (immune sera, week 9) respectively. Mucosal samples were collected at week 9. Nasal washes were collected by lavaging mouse nostrils repetitively with 250 µl PBS containing 0.05% Tween 20 (PBST). For tracheal washes, trachea from immunized mice were soaked in 250 µl PBST overnight. To collect lung lavage, individual mouse lungs were lavaged repetitively with 1 ml PBS. After a brief centrifugation (8000 rpm) for 10 min, supernatants were filtered through a 0.22 µm filter and stored at −80°C for further assays.

### Live virus challenge

Immunized mice were challenged with mouse-adapted PR8 H1N1 or Philippines H3N2 viruses 4 weeks after the boosting immunization (week 9). A dose of 40 LD_50_ (equal to 2000 infectious particles for PR8 and 1000 for Philippines) was used for challenge infection. Mice were lightly anesthetized by inhalation of isofluorane, and viruses in a volume of 25 µl PBS were administered into mouse nostrils. Mouse body weight and survival were monitored daily. Groups of infected mice were sacrificed at day 4 post-infection to determine mouse lung virus loads. Lung virus titers were determined as previously described [31].

### Antibody responses

The influenza virus-specific serum antibody endpoint titers, including serum IgG and subtypes (IgG1, G2a, G2b and IgG3) and mucosal secretory IgA (sIgA) and IgG, were determined by enzyme-linked immunosorbent assay (ELISA) as described previously [31], [39]. In brief, 96-well microtiter plates (Maxisorp immunoplate; Nunc Life technologies, Basel, Switzerland) were coated with 100 µl/well of inactivated PR8 virus (5 µg/ml) in coating buffer (0.1 M sodium carbonate, pH 9.5) at 4°C overnight. For serum IgG titers to the heterologous Philippines H3N2 virus, plates were coated with 100 µl/well of inactivated virus (5 µg/ml in coating buffer). The serum or mucosal samples were serially diluted two-fold stepwise and 100 µl were added to wells. Horseradish peroxidase (HRP)-conjugated goat anti-mouse IgG, IgG1, IgG2a, IgG2b or IgG3 antibodies (Southern Biotechnology) were used for the determination of serum IgG, IgG1, IgG2a, IgG2b or IgG3 titers, and HRP-conjugated goat anti-mouse IgA or IgG antibodies were used for mucosal IgA or IgG titers. Color was developed using the substrate TMB (Zymed Invitrogen) and the optical density at 450 nm was read using an ELISA reader (Bio-Rad). The highest dilution which gave an OD450 twice that of the naïve group at the lowest dilution was designated as the antibody endpoint titer.

### Virus neutralization and hemagglutination-inhibition (HI) assays

Neutralization was performed using MDCK cells as previous described [31]. Hemagglutination inhibition assays were carried out as described [45] with modifications: 8 hemagglutination units of PR8 or Philippines viruses were mixed with serially diluted receptor-destroying enzyme-pretreated serum samples in a volume of 50 µl and incubated at 37°C for 1 h. An equal volume of chicken blood cells (0.5%) was added and incubated at 25°C for 30 min. The HI titer is the reciprocal of the highest dilution that inhibits hemagglutitation.

### Cytokine ELISpot assays

Interferon gamma (INF-γ) and interleukin 4 (IL-4) secretion from immunized mouse splenocytes were evaluated using ELISpot kits (eBioscience, San Diego, CA) according to the manufacturer's instructions. Splenocytes (1×10^6^ cells) isolated from immunized mice were seeded into 96-well filtration plate (MultiScreen™-HA, Millipore) precoated with anti-mouse INF- γ or IL-4 antibodies provided in kits. Cells were stimulated with a mixture of two major histocompatibility complex I (MHC-I) PR8 HA peptides (IYSTVASSL and LYEKVKSQL), a pool of five MHC-II PR8 HA peptides (SFERFEIFPKE, HNTNGVTAACSH, CPKYVRSAKLRM, KLKNSYVNKKGK, and NAYVSVVTSKYN), or inactivated PR8 at a concentration of 10 µg/ml. Because differentiated CD4+Th1 cells recognize antigens presented by MHC I, and consequentially produce Th1 cytokines (INF- γ TNF-β), these cytokines activate cell-mediated immune reactions including cytotoxic reactions executed by CD8+T cells. Thus CD8+T cell responses is a consequence of CD4+Th1 activation and consistent with the later.

### Statistical analysis

Comparisons of multiple treatments among vaccinated groups were performed using a non-matching two-way ANOVA followed by Bonferroni's post test (for Fig. 1 and 6). Comparisons of single treatment among vaccinated groups were performed using a non-parametric one-way ANOVA followed by Bonferroni's multiple comparison post test (for Fig. 2, 3, 5 and 8). Comparison of survival curves were performed using the Log-rank (Mantel-Cox) test (Fig.7A and C). The analyses were done by using GraphPad Prism version 5.00 for Windows (GraphPad Software, San Diego California USA). P values of less than 0.05 (p<0.05) were considered to be statistically significant. P<0.05(*), P<0.01(**), P<0.001(***), P>0.05(n.s.).
